# Histopathological Diagnosis of Primary Central Nervous System Lymphoma after Therapy with Corticosteroids or Anticoagulants

**DOI:** 10.3390/cancers16061157

**Published:** 2024-03-14

**Authors:** Julia Feldheim, Marvin Darkwah Oppong, Jonas Alexander Feldheim, Ramazan Jabbarli, Philipp Dammann, Anne-Kathrin Uerschels, Oliver Gembruch, Yahya Ahmadipour, Cornelius Deuschl, Andreas Junker, Ulrich Sure, Karsten Henning Wrede

**Affiliations:** 1 Department of Neurosurgery and Spine Surgery, University Hospital Essen, Hufelandstraße 55, D-45131 Essen, Germany; 2 Department of Neurology, Division of Clinical Neurooncology, University Hospital Essen, Hufelandstraße 55, D-45131 Essen, Germany; 3 Center for Translational Neuro- and Behavioral Sciences, University Hospital Essen, Hufelandstraße 55, D-45131 Essen, Germany; 4 Institute of Diagnostic and Interventional Radiology and Neuroradiology, University Hospital Essen, Hufelandstraße 55, D-45131 Essen, Germany; 5 Institute for Neuropathology, University Hospital Essen, Hufelandstraße 55, D-45131 Essen, Germany

**Keywords:** primary central nervous system lymphoma, stereotactic biopsy, corticosteroid, histopathology, brain tumor

## Abstract

**Simple Summary:**

In most cases, it is impossible to differentiate primary central nervous system lymphoma (PCNSL) from other brain tumors or neuroinflammatory diseases without histological confirmation. In most differential diagnoses of PCNSL, preoperative treatment with glucocorticoids is administered. This treatment reduces the symptoms of PCNSL but can also disguise the histological diagnosis, which can lead to an incorrect or delayed diagnosis of PCNSL and postponed therapy. To obtain a broader database for evidence-based decisions in the management of patients with PCNSL, we retrospectively evaluated all patients biopsied and diagnosed with PCNSL at our institution over the last 16 years. Particular focus was placed on the influence of preoperative glucocorticoid therapy and the effect of anticoagulation and dual antiplatelet therapy on the validity of histological diagnosis.

**Abstract:**

In patients with primary central nervous system lymphoma (PCNSL), the choice of surgical strategy for histopathologic assessments is still controversial, particularly in terms of preoperative corticosteroid (CS) therapy. To provide further evidence for clinical decision-making, we retrospectively analyzed data from 148 consecutive patients who underwent surgery at our institution. Although patients treated with corticosteroids preoperatively were significantly more likely to require a second or third biopsy (*p* = 0.049), it was only necessary in less than 10% of the cases with preoperative (but discontinued) corticosteroid treatment. Surprisingly, diagnostic accuracy was significantly lower when patients were treated with anticoagulation or dual antiplatelet therapy (*p* = 0.015). Preoperative CSF sampling did not provide additional information but was associated with delayed surgery (*p* = 0.02). In conclusion, preoperative CS therapy can challenge the histological diagnosis of PCNSL. At the same time, our data suggest that preoperative CS treatment only presents a relative contraindication for early surgical intervention. If a definitive diagnosis cannot be made after the first surgical intervention, the timing of a repeat biopsy after the discontinuation of CS remains a case-by-case decision. The effect of anticoagulation and dual antiplatelet therapy on diagnostic accuracy might have been underestimated and should be examined closely in future investigations.

## 1. Introduction

Primary central nervous system B-cell lymphoma (PCNSL) is a subtype of extranodal non-Hodgkin lymphoma confined to the brain, leptomeninges, spinal cord and eyes without a systemic manifestation [1]. It accounted for approximately 1.9% (incidence of 0.43 per 100,000) of primary brain tumors in the United States of America between 2011 and 2015 [2], has shown a continuous rise in its incidence in the past few decades and can occur in immunocompetent or immunocompromised patients [3]. Therapeutically, it represents a significant challenge, with approximately only every second patient surviving the first 12 months after diagnosis [2].

**Figure 1:**
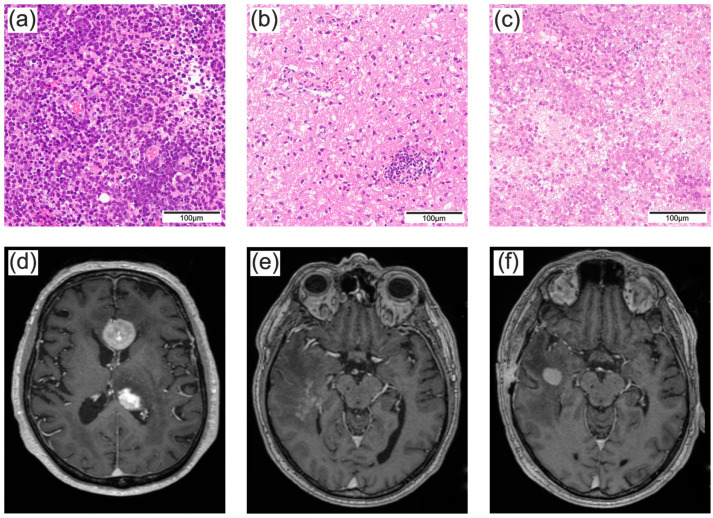
Consequences of corticosteroid treatment on PCNSL. Upper row: Exemplary hematoxylin-eosin staining of (**a**) untreated PCNSL, (**b**) PCNSL after high-dose CS treatment and (**c**) PCNSL after an interval of discontinued high-dose CS treatment. Bottom row: Exemplary T1-weighed contrast-enhanced magnetic resonance image of (**d**) untreated PCNSL, (**e**) PCNSL after high-dose CS treatment and (**f**) PCNSL after interval of discontinued high-dose CS treatment.

To verify the diagnosis of PCNSL, a histopathological diagnosis is essential [3]. However, the preferred surgical approach remains a controversial topic. A randomized phase III study showed a partial benefit of PCNSL resection for patients’ outcomes [4,5]. Subsequently, tumor resection might be a feasible option in some cases yet bears the risk of unintentionally resecting non-cancerous tissue or other brain tumors as they can often not be distinguished from PCNSL by clinical presentation or magnet resonance imaging (MRI) alone [6]. Therefore, stereotactic biopsy remains the most common surgical approach to verify the diagnosis of PCNSL today [3]. However, it deserves to be noted that diffusion-weighted imaging or magnet resonance spectroscopy may help to distinguish PCNSL from common differential diagnoses preoperatively [7]. One problem with the histopathological diagnosis of PCNSL that is not seen in the majority of other primary brain tumors is its strong response to corticosteroids (CSs). In cases of unclear intracranial mass lesions or autoimmune neuroinflammation, which are common differential diagnoses of PCNSL, high-dose CSs are a practical and frequently used preoperative treatment [8,9]. While CSs lead to a decrease in symptomatic tumor-associated edema without significantly affecting the tumor cells in most other brain tumors, they have an additional tumor-destructive effect in PCNSL. Via the glucocorticoid receptor, mitogen-activated protein kinases are activated, ultimately resulting in apoptosis of the PCNSL cells [10,11,12] (Figure 1).

While the CS effect may be desirable for the short-term improvement of neurological symptoms, it carries the risk of obscuring the diagnosis, thus significantly impairing the necessary therapy and the long-term outcome. Preoperative treatment with CS may even mimic inflammatory diseases in PCNSL [13]. Therefore, clinical guidelines recommend avoiding the application of CS when PCNSL is suspected and the patient’s clinical condition does not obligate CS treatment [3].

This dilemma leads to numerous problems in everyday clinical practice that need to be resolved by case-by-case decisions: should CS be used at the risk of prolonging diagnosis and causal treatment? Or should CS be avoided at the risk of depriving patients of effective symptomatic treatment? In addition, the feasibility of diagnosing PCNSL patients treated with CS may influence the decision on the optimal timing of surgery. Should the histopathologic diagnosis be rushed to allow earlier postoperative CS treatment? Or should surgery be delayed if patients have received preoperative CS to increase the chances of a definite histopathologic diagnosis? In clinical practice, CS will, if previously administered, most likely be discontinued before the surgery. However, clear guidelines on the ideal duration of the discontinuation are scarce.

In this study, we aimed to (1) compare the accuracy rate of the histopathologic diagnosis of PCNSL with or without preoperative CS treatment, (2) investigate the accuracy rate of histopathologic diagnosis after CS treatment depending on the time interval from the last CS application, (3) analyze whether different surgical approaches (e.g., stereotactic vs. open biopsy) provide an advantage for the histopathologic diagnosis of PCNSL (4) examine the informational benefit and potential disadvantages of preoperative diagnostics of the cerebro-spinal fluid (CSF), and (5) extrapolate possible implications for clinical decision making by retrospectively analyzing a collective of all patients that were diagnosed with PCNSL after a biopsy or resection at our institute between October 2008 and November 2022.

## 2. Materials and Methods

This retrospective single-center observational study comprised all consecutive patients who underwent a biopsy or the resection of PCNSL between October 2008 and November 2022. The study was conducted under the Declaration of Helsinki, was compliant with the Health Insurance Portability and Accountability Act of 1996 (HIPAA) guidelines, and was approved by the Institutional Review Board of the University of Essen (protocol code 22-11037-BO, date of approval: 9 January 2023). Suitable patients were identified by extracting a list of patients diagnosed with lymphoma after a biopsy or the resection of intracerebral tissue by searching an internal diagnostic database. Subsequently, patients were screened by two independent contributors (JF and KW) to determine whether they matched the inclusion criteria. All patients included were referred to us with an unclear brain lesion from other regional healthcare providers or departments after our interdisciplinary neurooncological tumor board had recommended neurosurgical intervention. Therefore, the preoperative diagnostics and therapy (including the application of CS) were highly heterogeneous. The histopathological diagnosis was classified by routine histology following the WHO criteria [14]. Only the cases with a histological confirmation of PCNSL diagnosis were included. PCNSLs that were diagnosed without a biopsy at our institution were excluded. Basic demographic and clinical data, such as the type of surgical intervention, CS treatment, suspected diagnosis and surgical intervention success, obtained within the routine clinical assessment framework, were collected from the hospital database via the manual extraction of data from the clinical documentation by two independent contributors (JF and KW). Preoperative imaging consisted of an MRI with at least T1- and T2-weighted sequences with and without a contrast agent as well as fluid attenuated inversion recovery (FLAIR) sequences and were independently evaluated by two experienced neuroradiologists.

Statistical analysis was carried out using IBM SPSS Statistics ver. 28 (IBM Corporation, Armonk, NY, USA). For demographic data, we reported numbers, percentages, medians and interquartile ranges. The normality was tested using the Shapiro–Wilk test. As the normal distribution was always rejected, differences between groups were determined using the Kruskal–Wallis test (KW) with a post hoc Dunn’s test (D-B)/the Mann–Whitney U-test (MWU-B) with correction of the significances according to Bonferroni for ordinal and metric variables or the chi-squared and Fisher’s exact test for categorical variables. Correlations were evaluated using Spearman’s correlation coefficient. The significance level was set to *p* < 0.05.

## 3. Results

### 3.1. The Majority of PCNSLs Were Diagnosed via Stereotactic Biopsies

In total, we identified 148 patients who underwent a neurosurgical procedure at our institution between October 2008 and November 2022 to confirm the histopathologic diagnosis of PCNSL. A slight majority of these patients were male (n = 88, 59.5%), and the median age in our cohort was 70 years (interquartile range: 60–76 years). In most cases, a stereotactic biopsy was performed to confirm the diagnosis (n = 96, 64.9%). In the remaining cases, sampling was performed via an open biopsy (n = 25, 16.9%), partial or complete tumor resection (n = 22, 14.9%) or other surgical procedures (n = 5, 3.4%), such as endoscopic biopsy. Most patients required only one biopsy to confirm the diagnosis (n = 138, 93.2%). Eight patients underwent a second surgery (5.4%) and two patients required three biopsies (1.4%). The tissue sample was most frequently taken from the frontal lobe (n = 66, 44.6%), followed by basal ganglia/thalamus (n = 21, 14.2%) and the occipital (n = 16, 10.8%), parietal (n = 13, 8.8%) and temporal lobe (n = 9, 6.1%). Nine patients had infratentorial biopsies (6.1%), and fourteen had other locations (9.5%), e.g., intraventricular. In the majority of cases (n = 109, 73.6%), the treating physicians already suspected PCNSL preoperatively, while 39 (26.4%) procedures were performed under different assumptions (e.g., suspected glioma). PCNSLs were highly proliferative with a median Ki67 staining of 80% (quartiles: 70–80%, not determined in two cases) and were surgically treated at a median of eight days after MRI diagnosis (4–17 days). Regarding the preoperative clinical status, 32 patients (21.6%) had ECOG status 0, 61 patients (41.2%) had ECOG status 1, 29 patients (19.6%) had ECOG status 2, 20 patients (13.5%) had ECOG status 3 and 6 patients (4.1%) had ECOG status 4.

### 3.2. Patients Treated with CS Had a Slightly Higher Risk of an Unsuccessful First Biopsy

Fifty-seven (38.5%) patients received preoperative CS treatment. Information on the type and dosage of the corticosteroid was rarely available as most patients were previously treated at external hospitals and referred for surgical biopsy after MRI diagnosis. In fifty of the 57 cases (87.7%), the diagnosis of PCNSL was confirmed after the first biopsy despite preoperative CS therapy. Five patients (8.8%) required two biopsies, and two (3.6%) required a total of three surgical procedures to confirm the diagnosis of PCNSL. In the 91 patients who had not received corticosteroids, the first biopsy was successful in 88 cases (96.7%), which was a slightly higher rate than in the CS group (*p* = 0.049, Fisher’s exact test), and only 3 patients (3.3%) required a second operation.

**Figure 2:**
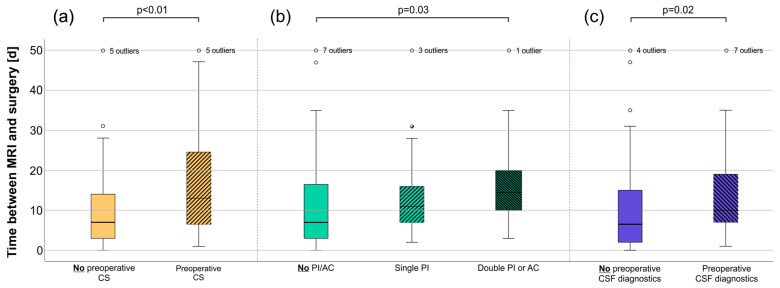
The time between the initial MRI and surgery. Boxplots show the period between MRI and surgery depending on whether patients were treated with (**a**) corticosteroids, (**b**) anticoagulation or platelet inhibition or received (**c**) diagnostics of the cerebrospinal fluid preoperatively. Abbreviations: CS, corticosteroids; PI, platelet inhibition; AC, anticoagulation; CSF, cerebrospinal fluid.

CS was, in median, discontinued 11 days (quartiles 4–15 days) before a successful first biopsy. In contrast, CS was discontinued 1, 4, 6, 8, 8 and 30 days (information missing for one patient) before surgery in the subgroup of patients who had an unsuccessful first biopsy after the administration of CS (Table 1). The time at which CS was discontinued preoperatively did not differ significantly between patients with successful and unsuccessful first biopsies (*p* > 0.05, MWU). Not surprisingly, patients that were treated with CS had a considerably longer time between the initial MRI and surgery (13 days [quartiles 6–26 days] vs. 7 days [quartiles 3–14.5 days], *p* < 0.01, MWU)—this time correlated to the time that CS was paused (r = 0.53, *p* < 0.01, Spearman, Figure 2a).

### 3.3. Anticoagulation or Platelet Inhibition Delayed Surgery and Was Associated with the Lower Success of the Biopsy

Next, we wondered whether patients treated with antiplatelet therapy (PI) or anticoagulation (AC) may have had a disadvantage, such as additional delays or impaired diagnostic accuracy due to a more cautious surgical approach. Although most of the patients were treated with neither PI nor AC (n = 104, 70.3%), 27 received single PI (18.2%), and 4 received double PI (2.7%). Twelve patients received AC (6/4.1% with coumarin derivatives, 1/0.7% with heparin, 4/2.7% with direct oral AC and 1/0.7% in combination with PI; no information was available for one patient). In 36 cases (24.3%), AC or PI treatment was entirely suspended before surgery. Twice (1.4%), one of multiple medications was continued, while the others were discontinued, and five patients (3.4%) required surgery while receiving AC or PI. Not surprisingly, the time between the MRI and surgery was significantly longer in patients treated with double PI or AC than in those treated with neither medication (*p* = 0.012, KW; *p* = 0.028, D-B; Figure 2b). Simple PI showed a non-significant tendency toward a longer preoperative time. The surgical methods chosen were independent of AC/PI (*p* < 0.05, Fisher’s exact test). On the other hand, the biopsy success rate was not. Five of the patients who were treated with neither AC/PI (4.8%) and one patient with single PI (3.7%) required a second biopsy, while the first biopsy was unsuccessful in four of the sixteen patients treated with double PI or AC (25%, *p* = 0.015, Fisher’s exact test). In addition, both patients with PCNSL diagnosed after the third biopsy were treated with PI/AC prior to surgery.

### 3.4. Cerebrospinal Fluid Diagnostics Were Independent of Previous CS Treatment

Preoperative diagnostics included a histopathological examination of the cerebrospinal fluid in almost half of our cases (n = 65, 43.9%). Twenty-nine of these (44.6%) showed no abnormalities, thirty showed mild (46.2%) and four severe pleocytosis (6.2%). Only twice (3.1%) did the pathologists find cells strongly suspicious for PCNSL. Fluorescence-activated cell sorting (FACS) was reported for 32 cases (21.6%). Of these, 26 were classified as unremarkable (81.3%), while FACS identified malignant or suspicious cells in 6 patients (18.8%). Though CSF diagnostics indicated suspicious results in a few of our cases, neither a pathological examination nor FACS was sufficient to reach a definitive diagnosis. CSF findings were independent of previous CS treatment, whether analyzed by a pathologist or FACS (*p* > 0.05, Fisher’s exact test). Interestingly, the time between the MRI and surgery differed between patients who underwent a preoperative lumbar puncture (median: 10 d [quartiles 6.75–19.5 d]) and those who did not (median: 6.5 d [quartiles: 2–15 d], *p* = 0.02; MWU; Figure 2c).

### 3.5. The Surgical Approach Did Not Influence the Accuracy of the Histopathologic Diagnosis and Depended Mainly on the Suspected Diagnosis

Interestingly, we did not observe a significant difference regarding successful tissue sampling between stereotactic and open surgery techniques (*p* > 0.05, Fisher’s exact test). However, the surgical methods chosen significantly differed depending on the patients’ CS treatment. In patients with preoperative CS, open biopsies (12/57, 21.1% compared to 13/91, 14.3%) and especially tumor resections (14/57, 24.6%, compared to 8/91, 8.8%) were more common. In contrast, patients not treated with CS underwent stereotactic biopsies (65/91, 71.4% compared to 31/57, 54.4%) and other interventions (e.g., endoscopic biopsies; 5/91, 5.5% compared to 0/57, 0%) more often. However, it should be noted that surgical intervention was primarily related to the preoperative assessment of whether PCNSL was suspected. Accordingly, most patients received a stereotactic biopsy (82/109, 75.2% compared to 14/39, 35.9%) or other surgical intervention (5/109, 4.6%, compared to 0/39, 0%) when PCNSL was suspected. In contrast, resections were more frequently chosen when other differential diagnoses were suspected (18/39, 46.2% vs. 4/109, 3.7%, *p* < 0.01, Fisher’s exact test). Open biopsies were equally frequent in both groups (17.9% vs. 16.5%). We also observed a slight tendency for patients with suspected PCNSL to have received CS less frequently (*p* > 0.05, chi-squared test).

## 4. Discussion

In line with previously published data, stereotactic biopsies were performed on most patients in the presented cohort [3,5]. The surgical approach was not associated with a successful histopathologic diagnosis, which is consistent with most previous studies. However, a small meta-analysis by Scheichel et al., found an inconclusive stereotactic biopsy rate of 8.6% after CS treatment and 1.9% without CS treatment [15]. In their analysis, open surgical procedures yielded a histopathologic diagnosis in all cases, largely similar to our observations [15]. We suspect that the presented cohort is too small to detect the marginal difference in diagnostic accuracy concerning surgical technique. 

Nevertheless, the choice of surgical approach remains an individual decision. The traditional view that the resection of PCNSL might even be an unfavorable prognostic factor has been challenged by Weller et al., who described the benefit of tumor resection [3,5,16,17,18]. More recent studies have supported the hypothesis that at least a subgroup of patients may benefit from tumor resection [19,20,21]. However, a retrospective analysis of the French oculo-cerebral lymphoma network, which included over 1000 subjects, showed no association between tumor resection and patient outcomes [22]. Still, stereotactic biopsies offer high diagnostic accuracy with comparatively low risk and may be preferred when there are no specific reasons for an open approach [23,24]. 

Interestingly, PCNSL was not the suspected diagnosis in one out of four biopsies. Most patients who underwent tumor resection also belonged to this group, suggesting that the decision for tumor resection instead of stereotactic biopsy was often made under the assumption of a different diagnosis. An alternative way to detect a PCNSL and an essential part of the diagnostic work-up is CSF cytomorphology and FACS. Previous studies reported that CSF diagnostics can only verify the correct diagnosis in a minority of cases [25]. However, we could neither confirm nor refute these observations, as patients whose PCNSL could be diagnosed without surgical intervention were excluded from our study by design. Notably, the detection rate of suspicious cells via FACS was superior to conventional cytopathology, as already hypothesized before [26,27,28] and was not influenced by previous CS treatment. However, it deserves to be noted that newer methods of CSF diagnostics, such as ultrasensitive circulating tumor DNA sequencing, show promising results that could set a new standard [29]. The time to surgery was significantly longer for patients who received a lumbar puncture. While it seems reasonable to perform preoperative CSF diagnostics because it might avoid surgical intervention, the decision should be weighed against the potential risk of a diagnostic delay [25].

One of the most essential clinical questions when PCNSL is a possible differential diagnosis is whether patients should be treated with CS and whether a prompt biopsy after CS administration is still useful. Our analysis showed a slightly lower success in confirming the diagnosis when CS was given before surgery. While this trend was to be expected, it is surprising that the success rate of the first biopsy was above 90% in both groups. Previous studies on this topic have provided mixed results: Brück et al. reported non-specific changes without clear evidence of PCNSL in 112 of 221 (>50%) biopsy specimens after CS therapy and concluded that CS should be avoided if possible [30]. In contrast, Porter et al. reported that only 8/68 (12%) of their cohort required a second biopsy after CS [31], suggesting that a biopsy under CS can be performed with high diagnostic accuracy if contrast enhancement is preserved on an MRI. It should be noted that 42 of their patients even underwent surgery during CS treatment [31]. Bullis et al. agree with the conclusions of Porter et al., while Haldorsen et al. report that repeat biopsies are required in 22% of patients [32,33]. 

Manoj et al. provide a differentiated observation by dividing their collective of patients depending on the duration of CS treatment. Similar to the reports from Brück et al., biopsies were inconclusive in up to 57% of their collective of patients that received CS for over one week [34]. A possible explanation for these discrepancies might, except from regional differences, lie in the high degree of interobserver variability, as reported by Önder et al. [35]. 

Velasco et al. examined additional aspects [36]. Similar to our collective, they reported that patients treated with CS required a second biopsy more frequently. However, the rate of an unsuccessful biopsy in both groups was low compared to other trials (12% vs. 4%). Secondly, they investigated the effect of pausing CS before surgery and stated that CS tapering and the duration of CS pause before surgery did not influence the likelihood of a false-negative result, which is supported by our observations [36]. Further, despite the high diagnostic accuracy after CS treatment, patients of their cohort who received CS suffered a diagnostic delay that was, once again, mirrored in our collective of patients [36]. The reasons for this delay cannot be answered with certainty. Still, one might speculate that a delay before surgery due to CS withdrawal, as is noticeable in the study by Velasoco et al., might play a significant role [36]. Also, clinical practice suggests that there is a collective of patients who are initially misdiagnosed and might receive CS as a therapeutic option before a biopsy is finally performed. 

In 2021, Scheichel et al. conducted a small meta-analysis that included data from their own cohort and several of the studies mentioned above. Interestingly, they found that CS had no effect on diagnostic accuracy in their own cohort but concluded that CS treatment led to an increased risk of inconclusive biopsies in the meta-analysis with an odds ratio of about 3.1. The success of the biopsy did not differ in the meta-analysis, regardless of whether CS was continued or paused [15].

Most immunocompetent PCNSL patients have neuropsychiatric deficits as their primary symptom. However, one in three patients shows signs of increased intracranial pressure, and one in six patients suffers from seizures at an early stage [16,37]. These symptoms can significantly impair the patient’s quality of life or even lead to life-threatening situations. They can also improve dramatically after the administration of glucocorticoids due to the reduction in brain edema and cytotoxic activity [38]. In our collective of patients treated preoperatively with CS, unsuccessful biopsies were more frequent. Nevertheless, PCNSL could be correctly diagnosed in over 90% of cases, and the diagnostic accuracy could be further increased by additional molecular diagnostics [39].

We hypothesize that the widespread concern about jeopardizing the histologic diagnosis via preoperative CS therapy may be overly cautious. When necessary, the pursuit of ideal biopsy conditions should not lead clinicians to withhold effective treatment from patients with suspected PCNSL and severe symptoms. Our data suggest that preoperative CS treatment does not present a definitive contraindication for early surgical intervention. If the first biopsy was unsuccessful and a second biopsy after CS therapy was discontinued, this yielded the correct diagnosis with few exceptions. However, like other authors, we could not establish a threshold for the ideal duration of the discontinuation of CS [15]. Therefore, if a definitive diagnosis cannot be made after the first surgical intervention, a second biopsy after the discontinuation of CS appears sensible but remains an individual case-by-case decision. 

A delay in diagnosis, e.g., due to a delay in surgery because of CS treatment, has been described as a negative prognostic factor in PCNSL [40]. In clinical practice, a common reason for delayed surgery is the use of anticoagulant medication. As expected, we observed a longer interval between the first MRI and surgery in patients treated with AC or dual PI, whereas single PI seemed to delay surgery only slightly.

However, to our surprise, a second or third biopsy was more frequently required to verify PCNSL in patients treated with AC or dual PI, and this effect might even outweigh the influence of previous CS treatment. Coagulation-related complications are not uncommon in patients with lymphoma, but to our knowledge, there is no known impact on tumor growth or diagnostic accuracy [41,42,43]. Although the surgical methods did not differ significantly between the two groups, it could be hypothesized that an increased risk of bleeding may have prompted surgeons to proceed more cautiously. Also, the delay before surgery may have led to the use of higher doses of CS. Apart from an unknown bias, we cannot exclude the possibility of statistical chance and therefore look forward to future studies to reproduce or refute this observation.

This study has some limitations that need to be considered: (1) Only patients whose PCNSL was diagnosed in our institute were included. Therefore, we cannot exclude the possibility that patients with PCNSL and an unsuccessful first biopsy may have died or refused reoperation and are therefore not included in the study population. (2) As patients were referred to us from regional centers, we cannot exclude a selection bias, which may have led us to overestimate the number of inconclusive biopsies. Data on the duration and dosage of CS treatment were also incomplete and could not be analyzed in more detail. However, as these observations relate to real-life situations where physicians often have to make decisions with incomplete data, we still consider them very valuable. (3) The study might be biased due to its retrospective and monocentric nature. (4) The study included patients treated over a decade and a half ago. Changes in clinical standards or procedures may also have affected the observations.

## 5. Conclusions

Preoperative treatment with CS for suspected PCNSL should be avoided, if possible, but should not be withheld from patients at all costs. Even after CS treatment, an early biopsy is justified as the diagnostic accuracy remains high. Therefore, our data suggest that preoperative CS treatment only presents a relative contraindication for early surgical intervention. If a definitive diagnosis cannot be made after the first surgical intervention, the timing of a repeat biopsy after the discontinuation of CS remains a case-by-case decision. An unexpected finding of our study was that treatment with AC and double PI affected the diagnostic accuracy (even when paused) and possibly even more significantly than CS treatment. These observations can currently not be finally explained and encourage further investigations.

## Figures and Tables

**Table 1:** PCNSL patients with unsuccessful first biopsy after CS therapy.

Patient	CS Paused before First Surgery [d]	First Surgery	CS Paused before Second Surgery [d]	Second Surgery	CS Paused before Third Surgery [d]	Third Surgery
1	30	STB	88	STB		
2	m.i.	OSB	≥259	STB		
3	4	STB	71	Resection		
4	8	OSB	19	OSB	48	STB
5	1	STB	11	STB		
6	8	OSB	21	Resection	141	Resection
7	6	STB	3	Resection		

Abbreviations: STB, stereotactic biopsy; OSB, open surgery biopsy; resection, (partial) tumor resection; m.i., missing information due to initial surgery at an external institution.

## Data Availability

Data are contained within the manuscript. Raw data are available upon reasonable request from the corresponding author. The data are not publicly available due to ethical restrictions.
